# Antioxidative Defense and Fertility Rate in the Assessment of Reprotoxicity Risk Posed by Global Warming

**DOI:** 10.3390/antiox8120622

**Published:** 2019-12-05

**Authors:** Costantino Parisi, Giulia Guerriero

**Affiliations:** 1 Comparative Endocrinology Lab, Department of Biology, University of Naples Federico II, 80126 Naples, Italy; cparisi@iimcb.gov.pl; 2 Laboratory of Zebrafish Developmental Genomics, International Institute of Molecular and Cell Biology, 02-109 Warsaw, Poland; 3 Interdepartmental Research Centre for Environment, University of Naples Federico II, 80134 Naples, Italy

**Keywords:** anthropogenic impact, biosentinels, fertility rate, global warming, reprotoxicity, ecosystem-based adaptation

## Abstract

The objective of this review is to briefly summarize the recent progress in studies done on the assessment of reprotoxicity risk posed by global warming for the foundation of strategic tool in ecosystem-based adaptation. The selected animal data analysis that was used in this paper focuses on antioxidative markers and fertility rate estimated over the period 2000–2019. We followed a phylogenetic methodology in order to report data on a panel of selected organisms that show dangerous effects. The oxidative damage studies related to temperature fluctuation occurring in biosentinels of different invertebrate and vertebrate classes show a consistently maintained physiological defense. Furthermore, the results from homeothermic and poikilothermic species in our study highlight the influence of temperature rise on reprotoxicity.

## 1. Introduction

It is now well known that, in the coming decades, the loss of biodiversity will have a dramatic impact on the life cycles of organisms [1,2,3]. Anthropogenic human activities and xenobiotics released into the environment such as metals, pesticides, herbicides, drugs, antifouling compounds, nanoparticles, and plastics directly influence migration, seasonal breeding, and reproduction [4,5,6]. These factors also have secondary deleterious effects contributing to climate change, as evidenced in global warming and ocean acidification, raising an alarm in the scientific community and government environmental agencies about reprotoxicity [7,8]. In particular, there is growing evidence on how higher temperatures can act together with even low chemical concentrations of pollutants to elicit significant effects, undermining the traditional risk assessment paradigm for establishing threshold conditions below which a compound is not considered a threat [5,9]. Furthermore, “environmental endocrinology”, focusing on active hormonal compounds, is bringing to light the hormonal mechanisms acting in response to changing environments [5,10,11].

In this context, climatic warming is causing alteration of the diurnal temperature range, as well as altered precipitation patterns [12]. These patterns have relevant effects on biodiversity, ecosystem function, and community structure. Moreover, physiology and life history traits such as thermal tolerance, growth rate, and reproduction are severely affected [13,14,15,16].

**Figure 1:**
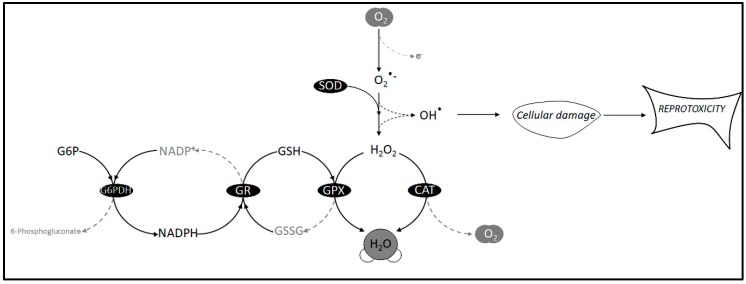
Schematic representation of the major antioxidant enzyme system countering free radicals in reprotoxicity. SOD, superoxide dismutase; CAT, catalase; GPX, glutathione peroxidase; GR, glutathione reductase; G6PDH, glucose-6-phosphate dehydrogenase; O_2_•^−^_,_ superoxide radicals; OH•, hydroxyl radical; GSH, reduced glutathione; GSSG, oxidized glutathione; NADPH, nitrate reductase; NADP^+^, nicotinamide adenine dinucleotide phosphate.

There are upper and lower temperature boundaries at which a species loses fertility. The combination of temperature fluctuations and the geographic distribution of these temperature boundaries, along with chemical pollution, some at low concentration, can lead to different adaptations to these factors among species [5,9,15,17,18]. These adaptations are closely dependent on how organisms use antioxidants to counteract oxidative stress due to rising temperatures. The overexpressed oxidants, specifically in the form of reactive oxygen species (ROS), are considered harmful to cells because they cause DNA damage in the form of mutations, base deletions, degradation, and single-strand scission, as well as disruption of cell membrane fluidity by peroxidation, all leading to apoptosis [12,19]. Indeed, antioxidant enzymes are the most important components in germ cells for countering reprotoxicity [19,20,21,22] (see Figure 1).

In the male species, germ cells lose antioxidant activity as they mature into haploid cells, with the most heat-sensitive stages of germ-cell development corresponding to the spermatids and the pachytene spermatocytes [22,23,24]. Extreme heat conditions in mammals commonly manifest as spermatogenic arrest, characterized by the onset of azoospermia, oligozoospermia, or teratozoospermia [25], owing to relatively low fertility arising from reduced semen quality [26]. In fact, acute heat stress in germ-cell populations causes elevated levels of DNA damage, leading to significant reductions in the success of embryonic development achieved following fertilization of oocytes once these cells mature to spermatozoa.

In the female germinal cells the oocytes characterized by a large cytoplasm have an antioxidant defense, which allows the oocytes to protect their genomic material as they transition through oogenesis [20,27]. In fact, a stringent surveillance is present in order to repair or eliminate oocytes with compromised genomic fidelity which could cause subfertility and infertility [20,28]. Heat stress during oogenesis compromises oocyte maturation, leading to alterations in follicular function, follicular growth, steroid secretion, and gene expressions [21,29,30,31]. The oocytes through the pre-ovulatory period are more susceptible to heat stress, and damage at this stage could reflect hormonal perturbations [32].

A change in global temperature and the exposure to sub-lethal concentrations of some chemical pollutants may influence ROS status and antioxidant defense systems in the physiology of organisms of different vertebrates and invertebrates. Thus, the primary objective of the present review is to summarize and collect information available in the literature on the effect of heat stress, resulting from global warming, on the oxidative damage, affecting fertility rates in invertebrate and vertebrate biosentinels which are useful for species protection and restoration in ecosystem-based adaptation.

## 2. Invertebrate Overview

Invertebrates are present in numerous ecological aquatic and terrestrial niches. Soil and water temperatures are key factors influencing their growth and survival, as well as indirectly influencing the availability of food. Most of the life-cycle traits are affected, such as weight, sexual maturity, reproduction, and life span [33]. Suitable biochemical markers are needed to monitor the reprotoxicity. Thus, the general approach is to study antioxidant activity in tissues and data extrapolation of oxidative damage occurring in the biosentinel. Effects of temperature fluctuation posed by global warming on antioxidant response (Table 1) and on fertility rate (Table 2) are briefly summarized.

### 2.1. Annelida

Phylum Annelida includes ringworms, earthworms, and leeches, which adapted to several ecological zones. Earthworms are an invasive species and arouse attention due to their beneficial roles in numerous soil processes and their sensitivity to xenobiotic contaminants and environmental stress [34,35].

At higher temperatures, some pesticides display a higher or lower effect in relation to their chemical activity. There is significant interaction between high temperature and biomarker expression activity in Annelida. In fact, in *Eisenia foetida*, Hackenberger et al. [34] showed that the temperature significantly decreased the enzymatic activity of glutathione *S*-transferase (GST) compared to a control group. Moreover, results on catalase (CAT) activity demonstrated that temperature, in conjunction with other abiotic factors, displayed a significant pattern in antioxidant defense. Thus, temperature–chemical variations assessed on Annelida demonstrate that high temperature may interact with the physiological response linked to toxic compounds.

As reported in Table 2, the high temperature toxicity is due to an enhanced metabolism, which may lead to greater pesticide uptake through the skin. Conversely, low toxicity at higher temperature could be due to reduced exposure concentration related to thermal instability of chemical compounds [58,59,60].

Evidence shows that even toxic contaminants, as a single predominant factor, are sufficient to inhibit or decrease the fecundity response. García-Torres et al. [48], using two species of Annelida, the redworm *Eisenia foetida* and the yellow-tail worm *Octolasion tyrtaeum,* demonstrated the effect of glyphosate in the soil. After timed exposure at different concentrations, the fertility rate decreased with increasing concentrations of glyphosate and, in both species, the number of hatched cocoons showed a statistically significant decrease.

### 2.2. Arthropoda

Arthropoda is the largest phylum that includes several living classes in variable environments, and it has to endure numerous challenges to survival and reproduction, including biotic and abiotic factors. Temperature is one of the most important abiotic variables affecting Arthropoda [36,41,42], and high temperatures can elicit in insects a variety of metabolic responses, including compensatory growth and ingestion of plant photo-oxidants [61,62].

Studies were conducted on different insects [38,39] and on a natural enemy of most pests such as predaceous coccinellids [40]. These are natural enemies of numerous small phytophagous insects and acarines and are considered beneficial natural enemies. They represent a third trophic level, which must cope with their own thermal stress.

Investigations on the Insecta, ladybeetle *Propylaea japonica*, show decreased survival of this phytophagous insect during increasing temperatures. A high expression of antioxidant cascade activity in the form of superoxide dismutase (SOD), CAT, and GST was found as a defense system to oxidative stress. Thus, the SOD activity exhibited significant activation with increased temperature at 39 °C, even though a decrease in SOD activity was observed at 41 °C. This pathway suggests that the activity of SOD might be involved in an adaptive response for overcoming high temperature and inducing ROS toxicity. Moreover, the elevated temperature could have overpowered antioxidant reserves, leading to the decrease at 41 °C. CAT and SOD work in stepwise oxygen reduction. In fact, SOD was significantly increased in response to heat stress in *Propylaea japonica* adults. GST activity was significantly affected by treatment temperature, suggesting that it is involved in the inactivation of toxic lipid peroxidation products accumulated due to oxidative damage induced by acute temperature stress [40].

In conjunction with toxic agents, abiotic factors such as high temperatures can seriously alter reproduction rates. In fact, toxic compound studies with heat-shock treatment on the common fruit fly, *Drosophila melanogaster* highlighted negative organophosphate compound effects on reproductive outcome. These studies revealed that these compounds caused a significantly reduced fecundity by alteration of reproductive performance in the exposed organisms, as well as a reproduction delay [49,63]. Moreover, a set of studies conducted on insects, clearly highlighted how temperature impacts fertility rate by affecting ejaculatory traits, sperm competition, mating frequency, egg size, and hatching rate (see Table 2).

### 2.3. Mollusca

Mollusca is the second largest phylum after Arthropoda. Several organisms belonging to this phylum also live on marine coasts and in freshwater. Studies on this phylum showed how temperature fluctuations affect the modulation of free-radical metabolism, leading to the activation of antioxidant defenses [43,44,45,47,64].

Temperature is a key environmental factor that interacts with and guides the physiology of marine poikilotherms such as Cephalopoda. They are very adaptable organisms which can adjust their biological and metabolic processes according to environmental and ecosystem changes [46,65].

Investigations on Cephalopoda, the common octopus *Octopus vulgaris*, showed how temperature fluctuations affect energy expenditure rates where their early life stages are related to ontogenetic metabolic differences and how the hypoxia resulted in their immediate vision loss [46,65,66,67].

The survival rate of Mollusca decreases around 30% under the near-future warming scenario, and an increase of only 3 °C above the average summer temperature shows deleterious effects on the early ontogeny. This may be caused by an increase in membrane permeability that leads to cellular injury mechanisms and to an enhancement of ROS activity leading to lipid peroxidation. In fact, during early developmental stages, such as embryogenesis, the antioxidant defenses are stimulated [46].

Mussels represent an important tool for biomonitoring environmental pollution; in fact, they are considered a sentinel organism, serving as a bioindicator to evaluate chemical pollutants in marine environments [68,69].

The Mediterranean mussel, *Mytilus galloprovincialis*, was used for monitoring heavy metal pollution with seasonal variations. Vlahogianni et al. [43] showed how SOD and CAT activity increased 2–3 times at the polluted sites, with high activity in the winter and spring time, showing a decrease in antioxidant defense enzyme activity in combination with high temperatures and toxicity.

Recent studies on the European flat oyster, *Ostrea edulis*, and on the eastern oyster, *Crassostrea virginica*, showed the direct effect of temperature on sperm production [55], gametogenesis, and sex ratio through the role of steroid hormones [56]. In fact, a correlation was found between estradiol and testosterone determined in developing gonads. However, a relationship among gonadal maturation, sex determination, and hormone concentration was not found. This study highlighted the role of temperature on gametogenesis and sex determination.

## 3. Vertebrate Overview

The vertebrate subphyla occupied and proliferated in numerous ecological aquatic, terrestrial, and airborne niches. Many and diverse factors influence their physiology and metabolism. Temperature seems to be one of the many factors that may influence reproductive biotic and abiotic control factors. The literature shows its influence on fertility directly by experiments done on endocrine tissues such as the hypothalamic–pituitary–gonad axis, or indirectly, on related tissues such as plasma or liver. Various species of vertebrates are model organisms for the purpose of monitoring effects of temperature fluctuation (Table 3) and reprotoxicity risk based on the environmental antioxidant response and effects on fertility rate (Table 4) posed by global warming.

### 3.1. Pisces

Fish are gill-bearing aquatic organisms, and most of them are poikilothermic. It is well known that environmental parameters such as temperature, pH, and dissolved organic carbon all influence the toxicity effect and fish physiology [70,71,72,73,74,75,76,118,119,120].

In particular, Braz-Mota et al. [76] investigated how the influence of temperature rise increases the chemical toxicity effect on the physiological responses of the Actinopterygii organism, the catfish *Hoplosternum littorale.*

The highest temperature assessed in their study (34 °C) showed a decrease in survival time in animals exposed to different toxic compound concentrations. Even the lower complexation of Cu^2+^ with higher temperature led to a decrease in the survival time.

Moreover, they observed that the ROS increase was dependent on the interaction of temperature and the Cu^2+^ concentration. SOD activity showed a concentration-dependent response, where higher compound concentrations induced a 2.7- to 4.4-fold increase in SOD, and, at the highest temperature, the increase was 3–4-fold after chemical exposure. Thus, high temperatures can lead to higher metal solubility and free metal ions, leading to stronger toxicity effects.

Fish reproduction is directly linked to higher water temperatures arising from climate change. Changes in environmental temperature are able to affect antioxidant defense, endocrine function, the advancing or retarding of reproductive processes, and gametogenesis and maturation, thereby reducing maternal investment and gamete viability. In fact, reduced egg size, fertility, and survival are partly a result of impaired 17β-estradiol (E_2_) secretion, hepatic vitellogenin synthesis, and sequestration during critical stages of vitellogenesis during oocyte development [94,121].

Pankhurst et al. [94] showed in Atlantic salmon, *Salmo salar*, how the exposure to elevated temperatures during gametogenesis affects gonadal steroid synthesis and hepatic vitellogenin production and reduces maternal investment and gamete viability. Likewise, Breckels at al. [95], using guppy fish *Poecilia reticulate,* showed how males raised at high temperature have shorter and slower sperm.

### 3.2. Amphibia

Organisms belonging to the Amphibia class are organized in three orders, Anura, Urodela, and Apoda. They occupy a wide variety of habitats including terrestrial, fossorial, arboreal, and freshwater aquatic ecosystems. The modern orders of amphibians are Anura (frogs and toads), Urodela (salamanders), and Apoda (caecilians).

Amphibians typically have a biphasic life cycle as larvae living in water and, generally, in the young phase, undergo metamorphosis from larva with gills to air-breathing adults. They usually use their own skin as a secondary respiratory surface, which is permeable to endogenous and exogenous substances whose potential harmful effects are contrasted by the combined action of keratins. This factor makes them particularly suitable as a biosentinel [122,123,124].

Studies on Amphibia Anura [78,79,81,82,125] and in particular on Ranidae, the Italian pool frog *Pelophylax bergeri,* showed how temperature fluctuations and pollutants can affect the antioxidant defense [123,126]. In fact, a large study (2011–2016) carried out along the coasts of Sarno (Italy) highlighted how a contaminated site produces radical content in *Pelophylax bergeri* skin tissues. It was observed that the radical content was statistically higher in the hotter period of the year (April), coinciding with the spermatogenesis phase, while a reduction in radical content was observed in the lower-temperature period (October–November) during the stasis phase. The same trends were observed at unpolluted control sites, showing the relationship between temperature rise and an increase in ROS content. Analyses on liver GST activity in polluted and control sites showed an increase of this enzymatic activity in April, with higher temperatures, compared to lower activity in lower-temperature periods such as October–November. This same pattern occurred in both polluted and control sites [78].

Analysis on testis DNA from *Pelophylax bergeri* performed during active spermatogenesis in the Italian warm season (April–May), in connection to environmental pollutant exposure, pointed out the role of nuclear poly(ADP-ribose) polymerases (PARP) activity in the fertility response. In fact, this effect is involved in the regulation of several cellular functions related to the maintenance of genomic integrity (DNA repair, gene amplification, apoptosis) and the expression and propagation of genetic information. Higher expression of PARP in the testis was found, highlighting the role of structural integrity in sperm motility and viability [96].

On the other hand, for amphibian females, a negative effect was observed between the female body condition and survivorship [127], whereby the artificial suppression of hibernation causes premature sexual reproduction. A study on the effect of temperature increase on life-history trait, such as fecundity, was conducted by Galloy and Denoël [97], showing how water temperature has an effect on the fecundity where only half as many eggs were laid at the highest temperature compared with the two lower-temperature treatments. In fact, the authors pointed out that, in nature, egg survival is 20 times lower than in the laboratory (3% vs. 64% in their experiments). Thus, if this fecundity is reduced by one-half, in the natural environment, these effects may be even more pronounced.

### 3.3. Reptilia

Poikilothermic organisms belonging to the Reptilia class include turtles, crocodilians, snakes, tuatara, and lizards. Lizards provide a good model to study the influence of climate warming on reproduction due to their behavior and physiology being highly dependent on environmental temperature. In fact, embryonic development, phenotypes, offspring number, and size can all be affected [16,83,84,100,128,129,130].

Analyses on Reptilia Squamata, such as on the ruin Lacertidae, *Podarcis sicula*, showed how combined factors, as well as high temperature and the activity of xenobiotics released into the environment, affect their physiology [5,98]. Specifically, the antioxidant defense activity by glutathione peroxidase 4 (gpx4), which was observed under steroid control [131,132], showed that over-expression in the testis is followed by an opposite trend in relation to the feedback of the brain (hypothalamus–pituitary)–gonad axis, indicating steroid control [5,85].

Oxidative radical content displays significant differences related to temperature increase. The gpx4 activity observed in the testis and brain showed how environmental stress as heat and/or pollution alters the expression of antioxidant defense. In fact, the ROS content in lizard brain was significantly lower than in the testis and displayed higher levels after being subjected to experimental conditions. Brain gpx4 expression showed statistically significant differences at different temperatures, but opposite trends in the testis and altered expression in both tissues, with evidence of testis morphological and DNA disruption. This could be explained by gpx4, which, as part of a survival mechanism, is switched on by exogenous stressors and counteracts stress-induced apoptosis [19,98].

A histological assessment of gonad tissue from *Podarcis sicula* in the Italian warm season, which coincides with the mating period, displayed well-developed seminiferous tubules and different stages of germinal cells including sperm. Drug exposure in the maturation stage during the hottest time period, with the aim of mimicking emerging estrogenic pollutants in the environment, showed a slowing of spermatogenesis processes into seminiferous tubules, a reduced lumen, and a depletion of sperm quantity. Testicular changes and spermatogenesis arrest at secondary spermatocyte levels were observed, highlighting a reorganization of testicular structure impacting male fertility [98].

Heat stress has serious consequences on female reproduction including offspring number and size, influencing embryonic development and offspring phenotypes.

Regarding oviparous animals, many studies focused on embryonic development at oviposition. In fact, studies on gravid females during warming treatments showed a faster rate of energy accumulation and a reduced incubation period, leading to earlier oviposition and increased embryonic mortality [99,100].

In addition, high-gestation-temperature treatments performed by Dubey and Shine [101] using the endangered Blue Mountain water skink, *Eulamprus leuraensis*, showed how lizard females gave birth two weeks earlier, to slightly smaller offspring.

### 3.4. Aves

Aves are homeotherm organisms, characterized by a very different behavior and ecosystem. Habitat and photoperiods have a strong influence on metabolism and life traits, which leads to these organisms being a migratory species.

Organisms belonging to this class are the most abundant tetrapods, with around 10,000 species present in many ecological niches. In fact, these habitats are characterized by several stress factors, as well as environmental, physiological, metabolic, and nutrition factors, which cause different biological responses in birds.

Biomonitoring studies on this class are relatively complex because seasonal physiological processes, such as molting, reproduction, and very high physical activity during migration cause variations in the redox state of wild and captive winged organisms [88,133,134,135].

Several studies were carried out on the Aves class with regard to antioxidant defense in response to high temperature [86,87,88,136]. Studies on the red junglefowl, *Gallus gallus*, exhibited physiological and metabolic changes. Chickens treated with high temperature (32–34 °C) showed a significant production of ROS, indicating mitochondrial respiratory chain activation, as determined by the activity of antioxidative enzymes such as SOD, CAT, and GSH, and the formation of malondialdehyde (MDA). In fact, an elevation in SOD activity that was dependent on the severity of the heat treatment indicated that the role of this enzyme in physiological response is linked to thermal stress [86,87], while no significant changes were detected in GPX activity [86].

Moreover, continuous heat stress derived from the surrounding environment results in biological changes concomitant with decreased disease resistance. In fact, Daghir [137] proved the negative impacts of stress due to high temperature on poultry.

The effect of heat stress on bird fertility, as well as on the chicken *Gallus gallus domesticus* [104] and the zebra finch *Taeniopygia guttata* [105], showed how high temperatures can affect the physiological and endocrine response, reducing sperm concentration and exhibiting altered sperm viability.

El-Tarabany [106] showed how high temperature can affect the Japanese quail, *Coturnix japonica*. Normal, moderate, and high environmental temperatures (23, 32, and 35.8 °C) were tested. Results showed a correlated decrease in body weight, feed intake, egg number, egg mass, fertility, and hatchability percentage.

In fact, these two latter deleterious consequences could be explained as the pulsatile gonadotrophin-releasing hormone generator frequency being disrupted by high environmental temperatures. Moreover, compromising of reproductive function was observed due to heat-induced impairment in the secretion of follicle-stimulating and follicle-luteinizing hormones in laying birds [106].

### 3.5. Mammals

Species belonging to the Mammalia class are homeotherms with body temperatures that range from approximately 35.8 °C to 39.8 °C. Indeed, regulation of core body temperature is a priority over several other physiological functions, and heat stress can affect the reproductive processes.

In species males, a suspended scrotum is characteristic of most mammals which have this apparatus outside the body cavity, with intratesticular temperature slightly lower than body temperature, as the site of spermatogenesis. In fact, there is an intricate thermoregulatory system in the testis for heat exchange [21].

On the other hand, for species females, oogenesis takes place in the ovaries, leading to oocytes. In fact, during follicular development, high estrogenic activity is dependent on the granulosa cells where the stimulation from follicle-stimulating hormone (FSH) binding to its receptors is essential [110].

Heat-induced oxidative stress in testes and ovaries occurs mainly via lipid peroxidation of the cellular membrane and mitochondria-derived ROS in the male, and by low estrogenic activity in the follicles in the female [107,110,138].

In fact, ROS formation is always followed by an upregulation of the antioxidant defense system, which protects tissues against cellular damage due to the scavenging activity of enzymes including SOD, CAT, and GPX [91,139].

Studies on testicular chemical stress using house mouse *Mus musculus* showed how oxidative damage was accompanied by decreases in SOD, CAT, and GPX activity [139], as well as in the scrotal heat-induced oxidative stress. In fact, it was observed that GSH concentration and activities of GPX, CAT, and T-SOD primarily decrease, then return to the initial levels after a period of recovery (seven days), indicating scrotal heat-induced oxidative stress in the testes [24].

Moreover, studies on elevated scrotal temperature in Mammalia, such as the house mouse *Mus musculus* testes [22,24,139], and in the pig *Scrofa domesticus* [114], showed how high temperature can lead to severe genetic and morphological damage. In fact, scrotal heat significantly induces DNA fragmentation in germ cells and spermatozoa, alters epididymal structure and epididymal sperm maturation, reduces the relative testis weight, and impairs steroidogenesis and lipid droplet accumulation observed in Leydig cells in mouse.

Further studies using shock/acute heat treatment (42 °C for 30 min/35 °C for 24 h) on the house mouse testes showed a lower concentration of spermatozoa with reduced viability and low motility, with impairment of spermatozoa integrity [22,108].

On the other hand, as for male gametes, heat stress can alter the development and function of oocytes. Evidence for these consequences comes from investigation on the house mouse *Mus musculus* testes, cows *Bos taurus*, wild goats *Capra aegagrus*, and pigs *Scrofa domesticus*, showing that hyperthermia reduces levels of gonadotropin receptors, estradiol plasma and follicular concentrations, aromatase activity, and luteinizing hormone (LH) receptor levels. It is especially noteworthy that heat stress induced a delay in ovulation with reduction of competence of germinal vesicle stage oocytes [27,30,110], increasing the numbers of small and medium follicles [29,115,116,117]. In addition, studies on the effect of paternal heat stress (36 °C for 24 h) on the development of embryos showed a significantly reduced embryo proportion. It was observed that protracted heat stress exposure (14–21 days) of mouse parents affects the development of embryos, leading to non-developed, abnormal, and dying/dead embryos [112,113].

## 4. Conclusions

The antioxidative physiological response and fertility rate play a central role in the distribution and abundance of natural populations and species. In the main classes of vertebrates and invertebrates, there are numerous reports showing the importance of oxidative stress due to a pollution event and/or temperature rise. The adverse effect of oxidative damage may affect gametogenesis, as well as embryo and larval development, leading to the loss of biodiversity. Moreover, damage in germ lines appears as a risk factor, leading to poor fertilization and impairment of pre-implantation embryos, thus increasing the risk of mortality in the offspring. Over the last two decades, much effort was made to decipher the role of antioxidants in reproductive health and fertility rate for the assessment of the reprotoxicity risk posed by global warming. In our update, we briefly summarized our recent progress and data from various studies performed between 2000 and 2019 on oxidative damage related to temperature fluctuations occurring in invertebrates and vertebrates, with the aim of developing strategic environment-based adaptation solutions. In addition, we phylogenetically highlighted the influence of temperature in homeotherms, as well as in poikiloterms, on increases in reprotoxicity and its effect on physiological antioxidant responses and fertility rate. In fact, 32.5% of our reviewed studies concerned invertebrates, of which 34% considered the male species, highlighting the effect of increased temperature on reproductive performance, in terms of fertility reduction, spermatogenesis alteration, and perturbation of male mating signals. Furthermore, 22% of the studies focused on invertebrate data on females, showing how the fecundity, hatching rate, and offspring success are altered by heat stress. The remaining 44% of the studies considered sexually immature invertebrate species. On the other hand, 67.5% of our data concerned vertebrates, of which 50% were relevant to the male species, showing DNA fragmentation in germ cells and spermatozoa, altered sperm viability, and low motility. Moreover, for female species, 36.5% of our data showed inhibition of ovarian activity and delay of follicular development, with a decrease in oocyte competence. Immature or not sexually identified species comprised 13.5% of the studies in this overview. In addition, we also showed how the deleterious effects of high temperatures affect not only parents, but also seriously affect the development of offspring by altering the development of embryos. Taken together, the data obtained by these investigations reinforce the importance of the biotechnological detection of antioxidants and fertility rate for the assessment and awareness of reprotoxicity risk posed by global warming. Furthermore, these tools include guidelines for mainstreaming ecosystem-based adaptation, enhancing resilience and providing evidence to help managers, communities, and decision-makers in their response to climate changes.

## Figures and Tables

**Table 1:** Data references for oxidative damage studies related to temperature increases occurring in invertebrates.

Phylum	Class	Order	Family	Genus Species	Temperature Max Reached (°C)	Temperature Increase (°C)	Impact of Temperature on Oxidants and Antioxidants	Gender	Ref.
**Annelida**	Clitellata	Haplotaxida	Lumbricidae	*Eisenia foetida*	25	5	GST ↓	nd	[34]
	Polychaeta	Phyllodocida	Nereididae	*Laeonereis acuta*	27	11	CAT ↑ GST ↑	nd	[35]
					28	12	CAT ↑ GST ↑		
**Arthropoda**	Insecta	Hymenoptera	Apidae	*Apis mellifera*	36.2	2.2	SOD ↓; CAT ↑; APOX ↓; H_2_O_2_ ↓	nd	[36]
					44.8	4.6	SOD ↓; CAT ↑; APOX ↓; H_2_O_2_	nd	
				*Apis cerana*	45	20	SOD ↑; POD ↑; GR ↑	M/F	[37]
		Lepidoptera	Crambidae	*Chilo suppressalis*	36	8	SOD ↑; ROS ↑; CAT	nd	[38]
					33	5	ROS ↑; CAT↑;		
					39	11	ROS ↑; CAT		
			Saturniidae	*Antheraea mylitta*	35	7	SOD ↑; CAT ↑; ASA ↑;	M	[39]
					40	12	GST ↑; LP ↑; SOD ↑; CAT ↑; ASA↑		
		Coleoptera	Coccinellidae	*Propylaea japonica*	35–41	10–16	CAT ↑; TAC ↑;	nd	[40]
					39–41	14–16	GST ↑; MDA ↑;		
					39	14	SOD ↑		
					41	16	POD ↑		
	Malacostraca	Decapoda	Portunidae	*Scylla serrata*	31.87	10.05	SOD♂ ↓; SOD♀ ↑; CAT♂♀ ↑; GPX♂ ↓; GPX♀ ↑; ASA♀ ↑;	M/F	[41]
	Maxillopoda	Sessilia	Archaeobalanidae	*Balanus balanoides*	29.6	0.5	SOD ↑; CAT ↑	nd	[42]
					30.1	0.4	GST ↑ SOD ↑; CAT ↑		
**Mollusca**	Bivalvia	Mytilida	Mytilidae	*Mytilus galloprovincialis*	20–25	11–14	SOD ↓; CAT ↓; LP ↑	nd	[43]
				*Perna viridis*	29.43	2.91	GST ↑; SOD ↑; CAT ↑; GR ↑; H_2_O_2_ ↑; LP ↑GSH ↑; ASA ↑	nd	[44]
					32.48	5.96	GPX ↑; SOD ↑; CAT ↑; GR ↑; H_2_O_2_ ↑; LP ↑; GSH ↑; ASA ↑		
		Ostreida	Ostreidae	*Crassostrea rhizophorae*	28	8	GST ↑; CAT↑; TOSC ↓	nd	[45]
					30	9	GST ↑; CAT↑; TOSC ↓		
	Cephalopoda	Octopoda	Octopodidae	*Octopus vulgaris*	21	3	GST ↑; MDA ↑	nd	[46]
	Gastropoda	Stylommatophora	Helicidae	*Helix aspersa*	21–22	2–3	CAT ↑; SOD ↑; GPX↑; GR ↓, GST ↓; G6PDH ↓	nd	[47]

ROS, reactive oxygen species; SOD, superoxide dismutase; POD, peroxidase activity; CAT, catalase; GSH, glutathione; GST, glutathione *S*-transferase; GPX, glutathione peroxidase; GR, glutathione reductase; G6PDH, glucose-6-phosphate dehydrogenase; TAC, total antioxidant capacity; ASA, ascorbic acid; APOX, ascorbate peroxidase; H_2_O_2_, hydrogen peroxide; LP, lipid peroxidation; MDA, malondialdehyde; TOSC, total oxyradical scavenging capacity. ↑ increase; ↓ decrease in enzymatic activity; nd, not determined. M, male; F, female; Max, maximum; Ref., reference.

**Table 2:** Data references for fertility studies related to temperature increases occurring in invertebrates.

Phylum	Class	Order	Family	Genus and Species	Temperature Max Reached (°C)	Temperature Increase (°C)	Impact of Temperature on Fertility	Gender	Ref.
Annelida	Clitellata	Haplotaxida	Lumbricidae	*Eisenia foetida* *Octolasion tyrtaeum*	22	-	Fertility rate reduction, decrease in hatched cocoons	F	[48]
Arthropoda	Insecta	Diptera	Drosophilidae	*Drosophila melanogaster*	37	13	Reduced fecundity, reproductive performance alteration, reproduction delay	M/F	[49]
		Rhynchota	Cimicidae	*Cimex lectularius*	34	12	Fecundity and offspring success alteration	F	[50]
					36	14			
					38	16			
		Hymenoptera	Megachilidae	*Osmia bicornis*	22–26	5	Perturbation of male mating signals and female choice	M	[51]
		Coleoptera	Chrysomelidae	*Callosobruchus maculatus*	33	8	Alteration of ejaculatory traits and sperm competition	M	[52]
		Coleoptera	Tenebrionidae	*Tribolium castaneum*	40–42	5–7	Sperm function and transgenerational damage	M	[23]
		Lepidoptera	Tortricidae	*Grapholita molesta*	35	11	Adult longevity, fecundity, egg size, and hatching rate	F	[53]
		Hymenoptera	Pteromalidae	*Anisopteromalus calandrae*	32–34	2–4	Alteration of male reproduction, sperm quantity	M	[54]
Mollusca		Ostreoida	Ostreidae	*Crassostrea virginica*	28	4	Decrease in sperm production and apoptotic bodies in testis	M	[55]
					32	8			
				*Ostrea edulis*	10	2	Alteration of gametogenesis and sex ratio	M/F	[56]
					14	6			
					18	8			
				*Crassostrea gigas*	25	7	Perturbation of gonadic differentiation	M/F	[57]
					28	10			

M, male; F, female.

**Table 3:** Data references for oxidative damage studies related to temperature increases occurring in vertebrates.

Phylum	Class	Order	Family	Genus and Species	Temperature Max Reached (°C)	Temperature Increase (°C)	Impact of Temperature on Oxidants and Antioxidants	Gender	Ref.
Chordata	Actinopterygii	Anguilliformes	Anguillidae	*Anguilla anguilla*	28	22	GST ↑; CAT ↑; GSH ↑; GPX ↑; TOSC ↓; GR ↓	nd	[70]
		Cichliformes	Cichlidae	*Geophagus brasiliensis*	38.2	0.8	CAT ↑; SOD ↓; GST ↓; GSH ↓;GT ↓	M	[71]
		Cyprinodontiformes	Fundulidae	*Fundulus heteroclitus*	23	17	GSH ↓; GPX ↑; LPO ↑	M/F	[72]
		Mugiliformes	Mugilidae	*Mugil cephalus*	28	22	GST ↑; CAT ↑; GSH ↑;GPX ↑; TOSC ↑; GR ↓	nd	[70]
		Perciformes	Moronidae	*Dicentrarchus labrax*	21	4.5	GPX ↓; AA ↓	M	[73]
			Sciaenidae	*Micropogonias furnieri*	24	12	GSH ↓; GST ↓; LP ↓;TAC ↑	nd	[74]
		Pleuronectiformes	Soleidae	*Solea senegalensis*	24	6.7	LP ↑; GST ↑; GPX ↑	nd	[75]
		Siluriformes	Callichthyidae	*Hoplosternum littorale*	34	5.0	ROS ↓	nd	[76]
					33	8	CAT ↑; GR ↑ GST ↑ LPO ↓	nd	[77]
			Loricariidae	*Loricariichthys anus*	24	12	GSH ↓; GST ↓; LP ↓;TAC ↑; GCL ↑	nd	[74]
			Pimelodidae	*Parapimelodus nigribarbis*	24	12	GST ↑; LP ↑;TAC ↑; GCL ↑	nd	[74]
				*Pimelodus pintado*	24	12	GSH ↓; GST ↑; LP ↓; GCL ↓	nd	[74]
	Amphibia	Anura	Ranidae	*Pelophylax bergeri*	21.8	4.3	ROS ↑; GST ↑	M	[78,79,80]
				*Rana ridibunda*	24.4	16.8	SOD ↑; CAT ↑, GPX ↓	nd	[81,82]
	Reptilia	Crocodylia	Alligatoridae	*Caiman yacare*	27.3	4.3	LPO ↑	M	[83,84]
			Crocodilidae	*Crocodylus johnstoni*	27.3	4.3	LPO ↑	M	[83]
				*Crocodylus porosus*	27.3	4.3	LPO ↑	M	[83]
		Squamata	Lacertidae	*Podarcis sicula*	31.6	17.8	ROS ↓; GPX ↓↑	M	[5,85]
	Aves	Galliformes	Phasianidae	*Gallus gallus*	32–34	8–10	SOD ↑; MDA ↑	M	[86]
					35	10	ROS ↑; SOD ↑; CAT ↑; GSH↑; MDA ↑	M	[87]
			Hirundinidae	*Hirundo rustica*	16.7	17.05	SOD ↓; GP ↓; GR ↓; G6PDH ↓; GST ↓; GSH ↓, LHP ↓	M/F	[88]
	Mammalia	Artiodactyla	Bovidae	*Bubalus bubalis*	39.46	12.26	SOD ↓; LPO ↓; NO ↑	F	[89]
		Rodentia	Muridae	*Mus musculus*	42	17	GSH ↓; GPX ↓; CAT ↓; SOD ↓	M	[24]
					40	15	GST ↑	M	[90]
					42	17	GPX ↑; GST ↑	M	[90]
					35	10	ROS ↑; GSH ↑; TBARS ↑	F	[91]
							FRSA ↑	F	[92]
			Cricetidae	*Myodes glareolus*	16.5	19.3	LP ↑	M	[93]

ROS, reactive oxygen species; SOD, superoxide dismutase; CAT, catalase; GSH, glutathione; GST, glutathione *S*-transferase; GPX, glutathione peroxidase; GR, glutathione reductase; GP, glutathione peroxidase; G6PDH, glucose-6-phosphate dehydrogenase; TAC, total antioxidant capacity; ASA, ascorbic acid; APOX, ascorbate peroxidase; H_2_O_2_, hydrogen peroxide; LP, lipid peroxidation; MDA, malondialdehyde; TOSC, total oxyradical scavenging capacity; GCL, glutamate–cysteine ligase activity; ACAP, antioxidant capacity against peroxyl radicals; NO, nitric oxide; TBARS, thiobarbituric acid-reactive substances; FRSA, free-radical-scavenging activity; ↑, increase; ↓, decrease in enzymatic activity; ↓↑, different patterns in various tissues; nd, not determined; M, male; F, female.

**Table 4:** Data references for fertility studies related to temperature increases occurring in vertebrates.

Phylum	Class	Order	Family	Genus and Species	Temperature Max Reached (°C)	Temperature Increase (°C)	Impact of Temperature on Fertility	Gender	Ref.
Chordata	Osteichthyes	Salmoniformes	Salmonidae	*Salmo salar*	22	4	Preovulatory shift inhibition, female reproductive development, reduced fertility, decreased egg survival	F	[94]
	Actinopterygii	Cyprinodontiformes	Poeciliidae	*Poecilia reticulata*	30	5; 7	Shorter and slower sperm	M	[95]
	Amphibia	Anura	Ranidae	*Pelophylax bergeri*	21.8	4.3	Testis DNA damage	M	[96]
		Urodela	Salamandridae	*Lissotriton helveticus*	22	14	Number Alteration of deposited eggs and oviposition period	F	[97]
	Reptilia	Squamata	Lacertidae	*Podarcis sicula*	31.6	17.8	Male fertility alteration, morphological defects	M	[98]
			Scincidae	*Scincella modesta*	24	4	Reduction of incubation period, increase of embryonic mortality and alteration of locomotor performance	F	[99]
					28	8	Maternally mediated changes in reproductive life history and induction of plastic responses in egg retention and offspring size	F	[100]
				*Eulamprus leuraensis*	20–33	3–16	Reduced incubation period leading to earlier ovoposition	F	[101]
				*Acritoscincus duperreyi*	22	1.80	Advanced embryonic development, increased hatching success	F	[102]
			Agamidae	*Amphibolurus muricatu*	33	3–6	Alteration of reproductive success	F	[103]
	Aves	Galliformes	Phasianidae	*Gallus gallus domesticus*	35	12	Fertility, sperm viability,	M	[104]
		Passeriformes	Estrildidae	*Taeniopygia guttata*	30	7	Male fertility, sperm concentration reduction, altered sperm viability	M	[105]
					40	17			
		Galliformes	Phasianidae	*Coturnix japonica*	35.8	12	Alteration of fertility and hatchability percentage	F	[106]
	Mammalia	Rodentia	Muridae	*Mus musculus*	35	2–7	DNA fragmentation in germ cell and spermatozoa, alteration of spermatogenesis, epididymal structure, epididymal sperm maturation and declines in sperm quality	M	[22]
					37–38	12–14	Impaired sperm motility and spermatozoa with plasma membrane changes within the cauda epididymidis	M	[107]
					42	9	Reduced sperm viability and low motility	M	[108]
					37–38	12–14	Reduced testes weights, increase in germ cell apoptosis, reduced sperm motility and higher percentage of spermatozoa showing membrane damage	M	[109]
					35	10	Ovarian dysfunction, estrogenic activity attenuation of growing follicles	F	[110]
					40	15	Disruption of developmental competence of germinal vesicle stage oocytes	F	[27]
					40	18	Developmental disruption of competence of the follicle-enclosed oocyte	F	[111]
					36	9	Damaged germ cells, impairment of embryos development in offspring	M	[112,113]
		Artiodactyla	Suidae	*Scrofa domesticus*	38	14	Alteration of quality and DNA integrity of spermatozoa	M	[114]
		Artiodactyla	Bovidae	*Bos taurus*	>25	<29	Inhibition of ovarian activity	F	[115]
					39.8	1.1	Oocyte competence decrease	F	[116]
					40.7	2	delayed effect on mediumsized and preovulatory follicles	F	[117]
					40.3	1.6	Delay of follicular development Increase of preovulatory plasma FSH	F	[29]
		Artiodactyla	Bovidae	*Capra aegagrus*	36	11	Growth ovulation suppression, decrease of estradiol synthesis activity in the follicles	F	[30]

M, male; F, female.
